# Open source all-iron battery 2.0

**DOI:** 10.1016/j.ohx.2020.e00171

**Published:** 2021-01-02

**Authors:** Dipak Koirala, Nicholas Yensen, Peter B. Allen

**Affiliations:** University of Idaho, United States

**Keywords:** Battery, Electrochemical cell, Rechargeable, Power, Energy, Renewable

## Abstract

In this work we present significant improvements to the open-source all-iron battery. We show higher power density and simpler fabrication. We also show a more reproducible procedure for preparing the electrolytes. The results are a highly rechargeable electrochemical cell based on iron, chloride, sulfate, and potassium ions in water at near-neutral pH. The cell is stable for thousands of cycles. It displays modest energy density consistent with the previous all-iron battery. The current is improved by a factor of 10 to a practical level of 500 mA/L and is able to deliver a maximal power of 250 mW/L. While this is modest performance compared to commercial rechargeable batteries, its low cost, simple synthesis, and safe manufacturing may make it suitable for storing renewable energy.

## Graphical abstract

**Figure:**
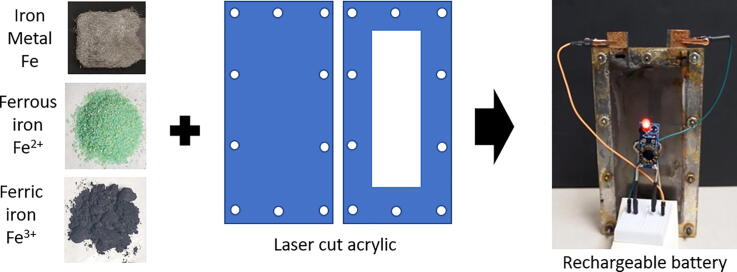

Specifications table

**Table:** 

Hardware name	*Open source Iron Battery 2.0*
Subject area	• Chemistry and Biochemistry
Hardware type	• *Electrical engineering and computer science*
Open Source License	*CC BY-NC-ND 4.0*
Cost of Hardware	*$4.58 - $13.74 for cells of 8 ml each; Cost of set-up $ 400 (to build 50 cells of 8 ml each)*
Source File Repository	https://doi.org/10.17605/OSF.IO/YV2E6

## 1 Hardware in context

Inexpensive, safe energy storage has many applications. Renewable energy can only displace a percentage of fossil fuel energy unless it can be efficiently and cost-effectively stored [1]. Lithium-ion batteries have emerged as the dominant energy storage system for mobile applications, but they have safety [2] and cost issues [3]. For stationary applications, it may be advantageous to move to a cheaper, safer, but lower energy density chemistry. We demonstrated a small-scale all-iron battery [4]. This cell was highly rechargeable with modest but useful energy density suitable only for low-power applications. We here report an improved version of this chemistry with similar energy density and much higher power density as well as a more convenient form factor.

Several all-iron batteries have been demonstrated in the literature and at large scale for commercial applications. For a recent summary, see Anarghya et al. [5]. Such batteries are often implemented as flow batteries [6], [7]. Flow batteries have the advantage of decoupling the energy capacity (determined by the size of the electrolyte tank) from the power capacity (determined by the size of the flow cell). This comes at the cost of relatively complex plumbing and pumps.

The all-iron battery presented here is a conventional battery and not a flow battery. Although the chemical reactions that move and store electrons are the same (i.e., the oxidation of Fe and the reduction of Fe^3+^), the physical design is much simpler. Rather than using a high-performance flow cell to achieve practical power levels, our approach is to modify the anode and cathode materials to achieve useful energy and power performance.

## 2 Hardware description

**Fig. 1:**
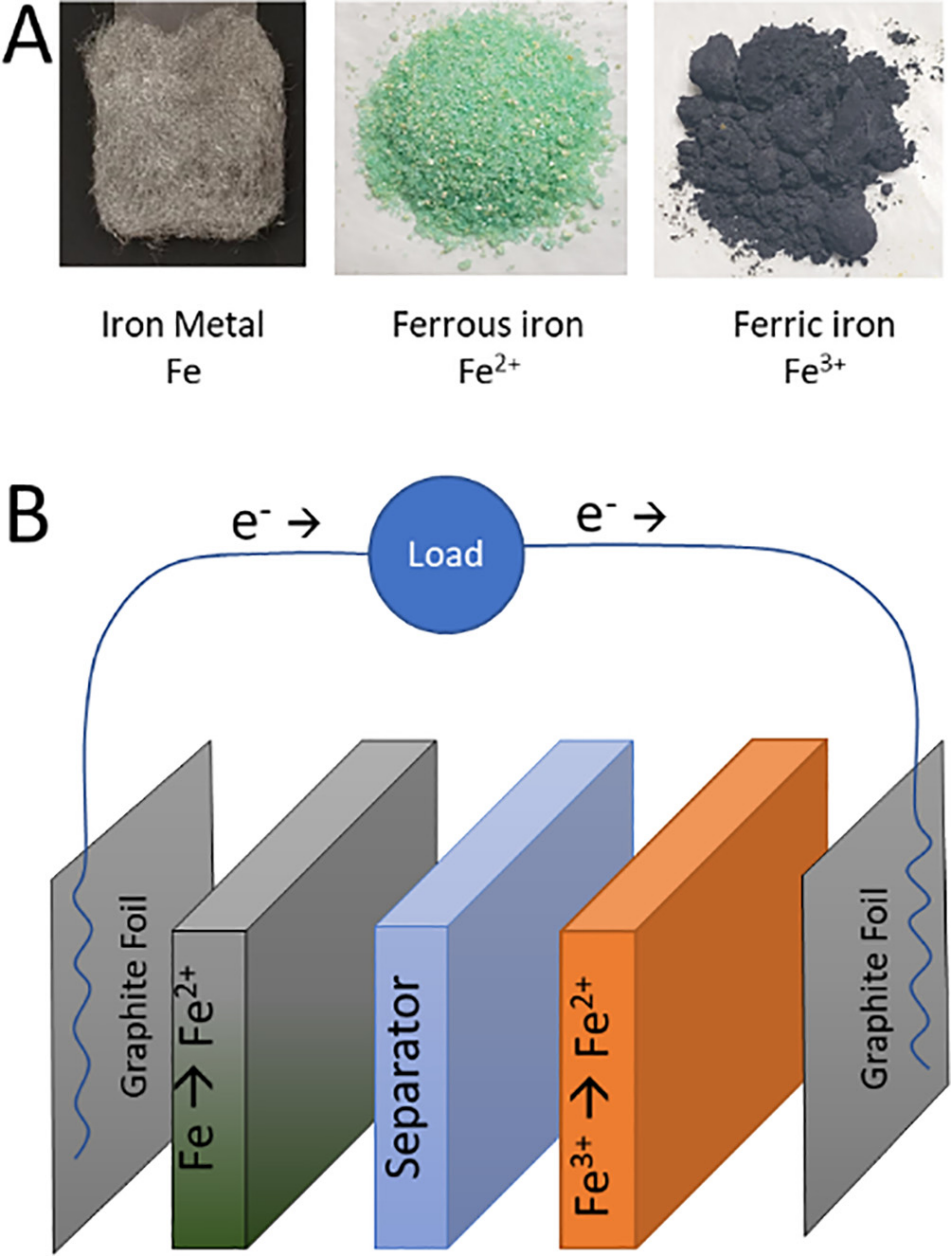
Overall battery design and function. (A) Images show the three active forms of iron. (B) A schematic show how the overall battery construction and current flow.

We describe a design for an energy storage battery with an iron-based anode and cathode. The overall strategy is shown in Fig. 1. Iron metal is oxidized to ferrous iron at the anode while ferric iron is reduced to ferrous iron at the cathode allowing electrons to flow. This system provides high rechargeability and customizability, but the power and energy density are low compared to commercial batteries. We also describe the simple synthesis of a suitable separator membrane made from cellulose acetate impregnated paper. This separator keeps the anode and cathode chemistry separate but allows ion transfer. The area of the polymer sheet determines the maximum current. For more power, the battery should be thin with a large surface area. If less power and more energy capacity is needed, the battery can be thicker with a smaller separator membrane area (i.e., a thicker acrylic plastic or multiple layers can be used).

**Fig. 2:**
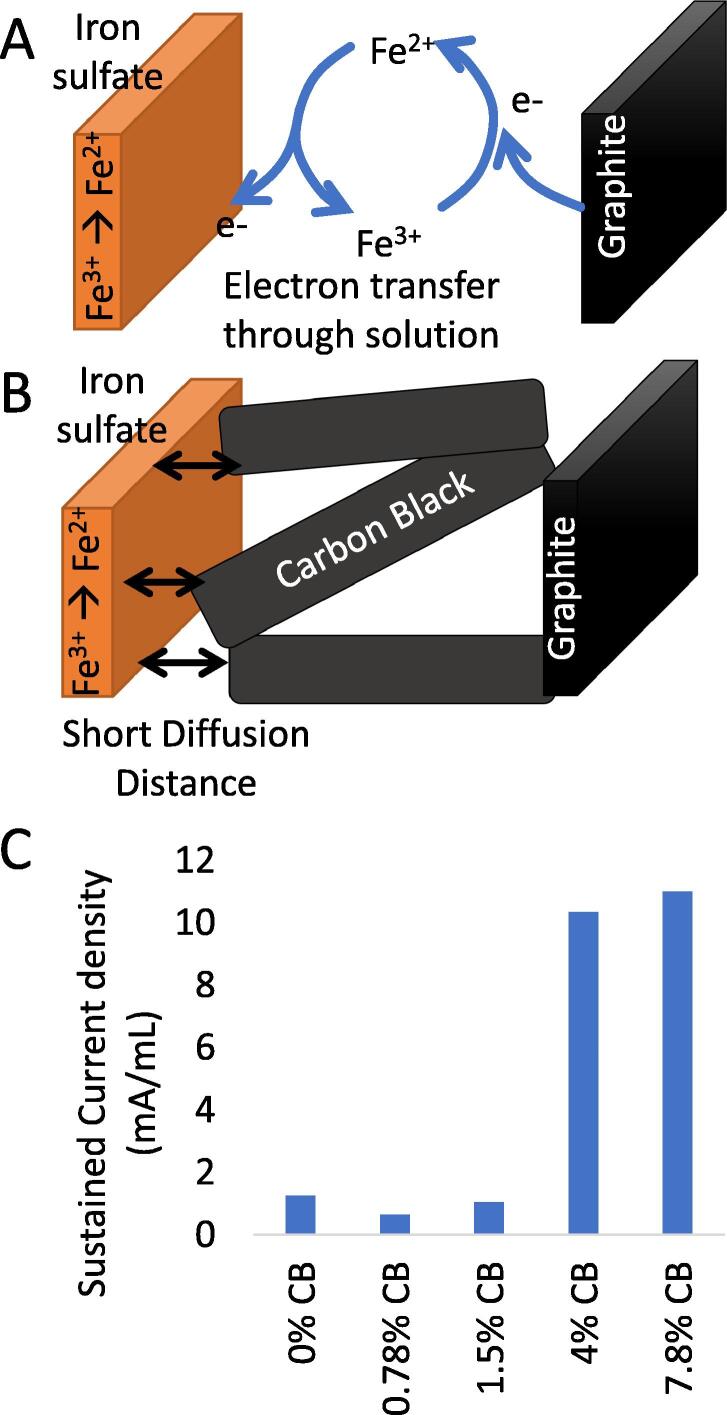
Ketjen black conductive particles improve battery performance. (A) Schematic shows how electrons diffuse through electrolyte. (B) Schematic shows how hypothetical effect of conductive carbon to shorten diffusion distance. (C) Graph of Sustained current vs % (w/w) of conductive carbon.

Our primary improvement to the original open-source all-iron battery is to increase the current density. The original formulation [4] was a mix of iron chloride and potassium sulfate adjusted to pH 7.5 with sodium hydroxide. Raising the pH causes a solid to precipitate. The conductivity of the precipitated particles is low and the concentration of iron ions in solution is also low. This limited the battery’s maximal electrical current. We determined that the maximal discharge and charge current could be significantly increased by adding conductive carbon. By decreasing the distance between the carbon electron conductor (originally, carbon felt) and the iron precipitate, we were able to increase power density. Adding ketjen black [Conductive Carbon] to the anode and cathode pastes allows electrons to migrate to within a much shorter distance of the active material through a conductive matrix (see Fig. 2) We added a range of concentrations of Ketjen black [conductive carbon] to the precipitate and determined that 4% carbon by mass was sufficient to reach near-optimal performance. More than 4% carbon means that there is less room for iron and so higher concentrations are not recommended.

**Fig. 3:**
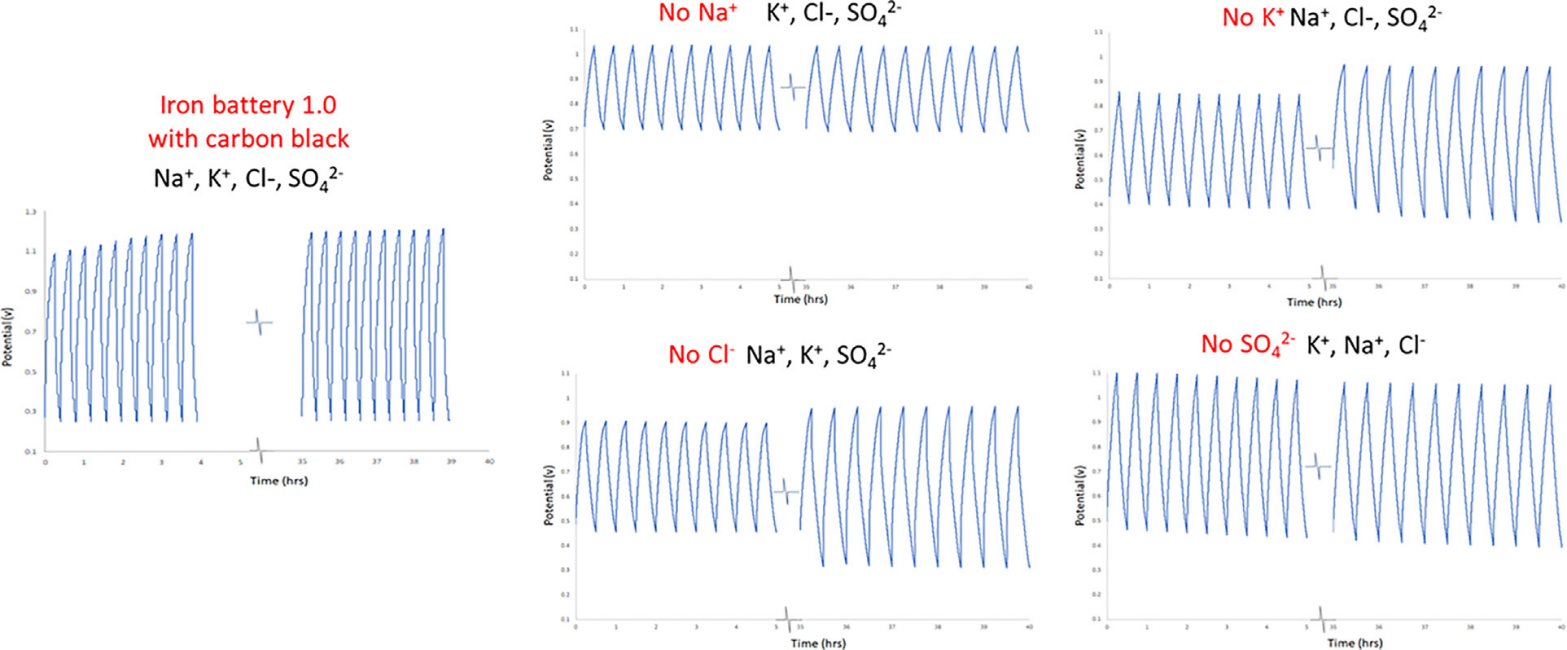
Optimization of cell chemistry includes cyclic potentiograms of each of four formulations with original iron chemistry (Iron Battery 1.0, at far left). In each case one ionic component (Na^+^, K^+^, Cl^−^, or SO_4_^2–^) was eliminated. The graphs show potential as a function of time during the first 10 and last 10 of 500 total cycles. Consistency in the charge-discharge profiles indicates stability.

As a secondary improvement we sought to simplify the original iron battery chemistry. In the original chemistry, the iron salt solutions contained dissolved iron, sodium, potassium, chloride and sulfate ions. This is shown at left, using the previous best membrane. The result was a stable chemistry with a large voltage drop (Fig. 3, far left). We replaced the sodium polyacrylate membrane with an improved membrane (cellulose acetate drop cast on printing paper). This improved the power density resulting in less internal resistance and a smaller voltage drop during discharge. We also systematically eliminated each component to determine which were necessary. Sulfate, chloride and potassium ions are necessary to generate a high performance, rechargeable cell. Only the removal of the sodium ions was tolerated with high performance (see Fig. 3, No Na^+^). Eliminating any of the other ions resulted in cell degradation and a changing charge–discharge profile. The newest formulation includes iron chloride and potassium sulfate precipitated with potassium hydroxide. Relative to the original chemistry, the new formulation is slightly simpler. However, depending on availability of materials, other formulations are possible (e.g., the use of ammonium iron sulfate) so long as the appropriate ions are dissolved in solution prior to precipitation.

This hardware could be useful in any context where a safe, inexpensive battery would be an advantage. The ability to use an open source energy storage solution could complement open source hardware projects in many fields:

• Storage of renewable energy from an open source wind turbine [8]
• Storage of renewable energy from an open source solar farm [9]
• Open source environmental data loggers [10]
• Open source weather stations [11]

## 3 Design files

Design Files Summary:

**Table:** 

Design file name	File type	Open source license	Location of the file
*Battery Design*	*CAD file in .svg format*	*CC BY-NC-ND 4.0*	doi.org/https://doi.org//10.17605/OSF.IO/YV2E6

*Battery Design:* The design files are included online at https://doi.org/10.17605/OSF.IO/YV2E6. The specified design includes a cell with external dimensions of 165 mm × 85 mm × 5 mm and an internal active volume of 9.6 ml. The cell is assembled from four acrylic parts and held together by a combination of solvent welding, glue and machine screws.

## 4 Bill of materials

The following is for 3 cells containing 8 ml each of the electrolyte pastes.

**Table:** 

Designator	Component	Number (per 3 cells of 8 ml each)	Cost per 8 ml cell - USD ($)	Cost per unit - USD ($)	Total cost - USD ($)	Source of materials	Material type
Fe Salt 1	Ferrous Chloride (FeCl_2_)	3.97 g	$0.25 $1.11 $0.66	$0.19/g $0.84/g $0.50/g	$47.90 $21 $49.95	Alfa-Aesar (A16327) CPLabSafety Amazon (B00QG9I3SK)	Inorganic
Fe Salt 2	Ferric Chloride (FeCl_3_)	3.24 g	$0.08 $0.04 $0.14	$0.08/g $0.035/g $0.13	$39.10 $17.60 $12.95	Alfa Aesar (A16231) Amazon (B00DYOA85Q)ebay	Inorganic
Salt	Potassium Sulfate (K_2_SO_4_)	6.97 g	$0.08 $0.23	$0.034/g $0.10/g	$34.40 $5.09	Alfa Aesar (A13975) ebay	Inorganic
Base	Potassium Hydroxide (KOH)	8.42 g	$0.13 $0.42 $0.28	$0.047/g $0.15 $0.10/g	$23.70 $15.31 $5.09	Alfa Aesar (A18854) Amazon (B07JVVTP56) ebay	Inorganic
Fe Metal	Steel Wool (Fe)	0.44 g	$0.01	$0.02/g	$3.78	Amazon/Walmart (B074MDTWQR)	Metal
	Cellulose Acetate	0.45 g	$0.07	$0.48/g	$11.92	Fisher-Scientific (AC177780250)	Organic
	Nafion	45 µL	$0.04	$2.46/ml	$61.50	Alfa Aesar (42118)	Organic
	Ethylene Glycol	67.5 µL	$0.01	$0.074/g	$18.60	Alfa Aesar (A11591)	Organic
	Printing Paper	1 pcs	$0.01	$0.013/sheet	$6.44	Amazon (B0050MRBA0)	Composit
Conductive Carbon	Ketjen Black EC-600JD	1.20 g	$0.43 $0.52	$1.07/gm $1.31/gm	$60 $65.50	Ebay Ebay	Inorganic
Housing	Arcylic sheet	6 pcs (5″ × 7″) Or, 1700 cm^2^	$4.40 $1.7	$2.20/pcs $0.003/cm^2^	$21.99 ~$30	Amazon (B081B15HL4) Local Hardware Store	Composite
Glue	Clear Seal	~6 ml	$0.05	$0.025/ml	$3.97	Amazon/Walmart (B001T8UDOU)	Composite
Elec 1	Graphite sheet	450 cm^2^	$0.15 $1.12	$0.001/cm^2^$0.008/cm^2^	$11 $23	Alibaba Amazon (B07K8Y4269)	Semi-conductor
	Methylene Chloride	6 ml	$0.11	$0.057/ml	$28.40	Alfa Aesar (L13089)	Organic
	Copper tape	30 cm^2^	~$0.50	–	$2.63	Walmart	Metal
	Nuts and bolts	30 pcs	$5 $1	$0.5/pc $0.1/pc	$15	Walmart Local hardware store	Metal/Alloy/Plastics
Totals (Lowest) $4.58/Cell (8 ml) Totals (Highest) $13.74/ Cell (8 ml)							

*Above mentioned prices are exclusive of local tax and shipping.

## 5 Build instructions

**Fig. 4:**
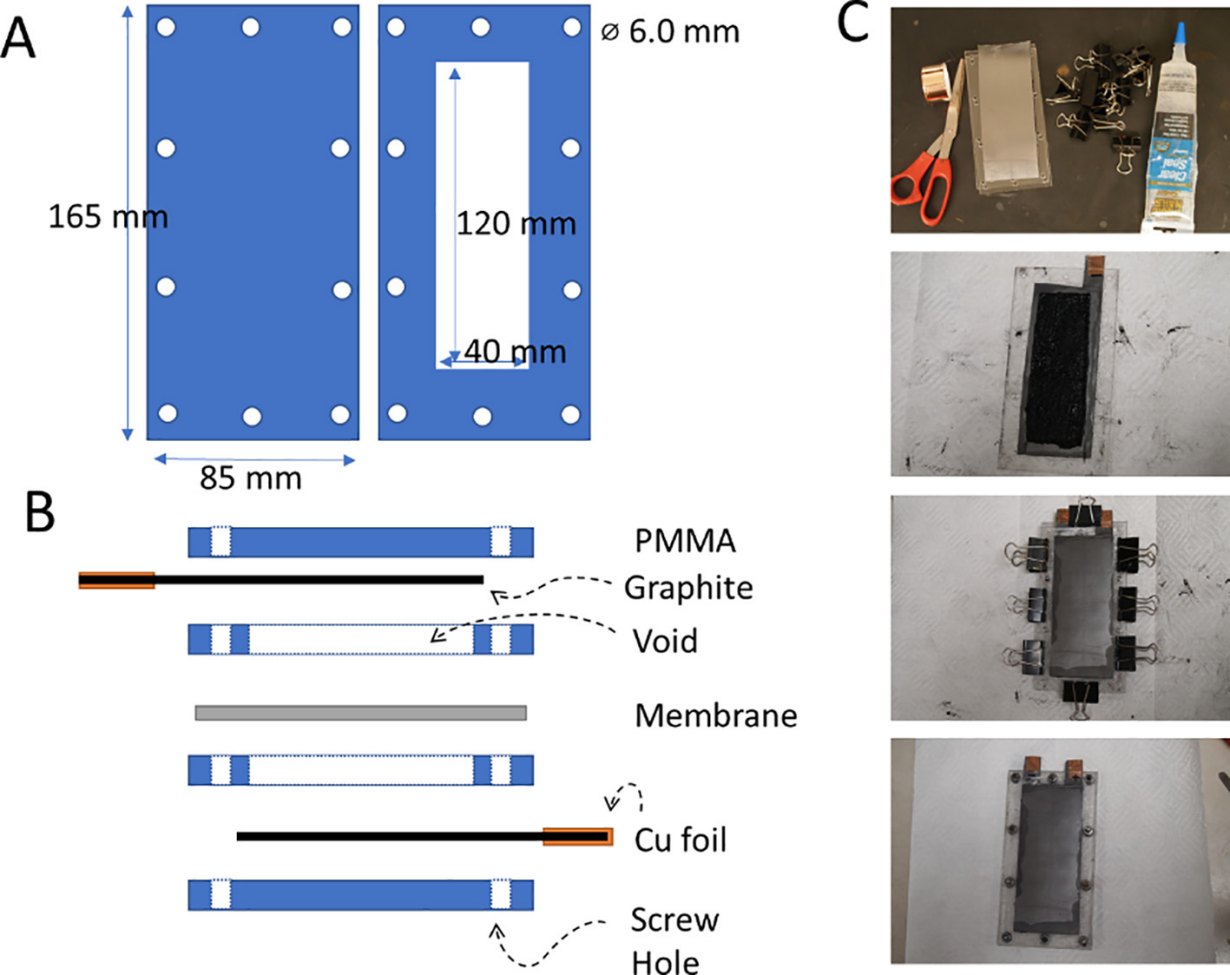
Cell design and construction. (A) CAD design of cell housing. (B) Schematic of cell assembly. (C) Photograph of image of materials and selected steps of cell assembly.

Short build instructions are as follows (detailed build instructions are included as Supplemental material). The cell housing was laser-cut from acrylic plastic (using a suitable CO_2_ laser cutter such as our BossLaser 80 W or acquired from a commercial service like Ponoko). The design can be adjusted to a wide range of dimensions. The CAD designs for the laser cut acrylic plastic (PMMA) are shown in Fig. 4A. Two copies of each part should be made. The internal enclosed volume determines the energy storage capacity. The surface area determines the power.

Graphite foil [Elec1] was cut to shape with tabs for making an electrical connection with alligator clips. The graphite foil was glued to a flat acrylic sheet using clear seal [Glue]. A second acrylic sheet was cut with a central hole slightly smaller than the graphite foil and glued to the first. The design leaves an open cavity for active material (i.e., either anode electrolyte paste or cathode electrolyte paste) as illustrated in Fig. 4B. A separator membrane was prepared by dissolving cellulose acetate in acetone, applying the solution to paper along with an optional small volume of Nafion, and allowing to evaporate (see Fig. S.2).

Electrolyte paste was prepared by dissolving iron chloride (ferric for cathode and ferrous for anode) in water at the appropriate proportions. To this, a measured quantity of potassium hydroxide solution was added to reach pH 7.5. To the resulting precipitate, ball-milled ketjen black [conductive Carbon] was added to reach 4%. The anode cavity was filled with finely divided steel wool [Fe Metal] and then packed with anode paste. The separator membrane was placed over the anode cavity. The cathode cavity was filled with cathode paste, then the cathode assembly was pressed over the separator membrane. The full assembly was then fastened together with machine screws at the perimeter and sealed by solvent welding the acrylic plastic with methylene chloride. Copper tape was used to reinforce the graphite foil tabs which extend beyond the edge of the acrylic. As assembled, the cell is in the charged state and is ready to generate electrical current.

## 6 Operation instructions

Once the battery is assembled it can be discharged and charged like any battery or electrochemical cell. This can generate the necessary electric current for a given electronic device. For discharge, the battery can be used to power any direct current electrical device that draws less than the maximum current for the cell (4.5 mA for the 8 ml cell). Multiple cells can be wired together in series or in parallel to generate higher voltage or current. For charging, we recommend a constant, regulated charging voltage of 1.1 V per cell in series.

**Fig. 5:**
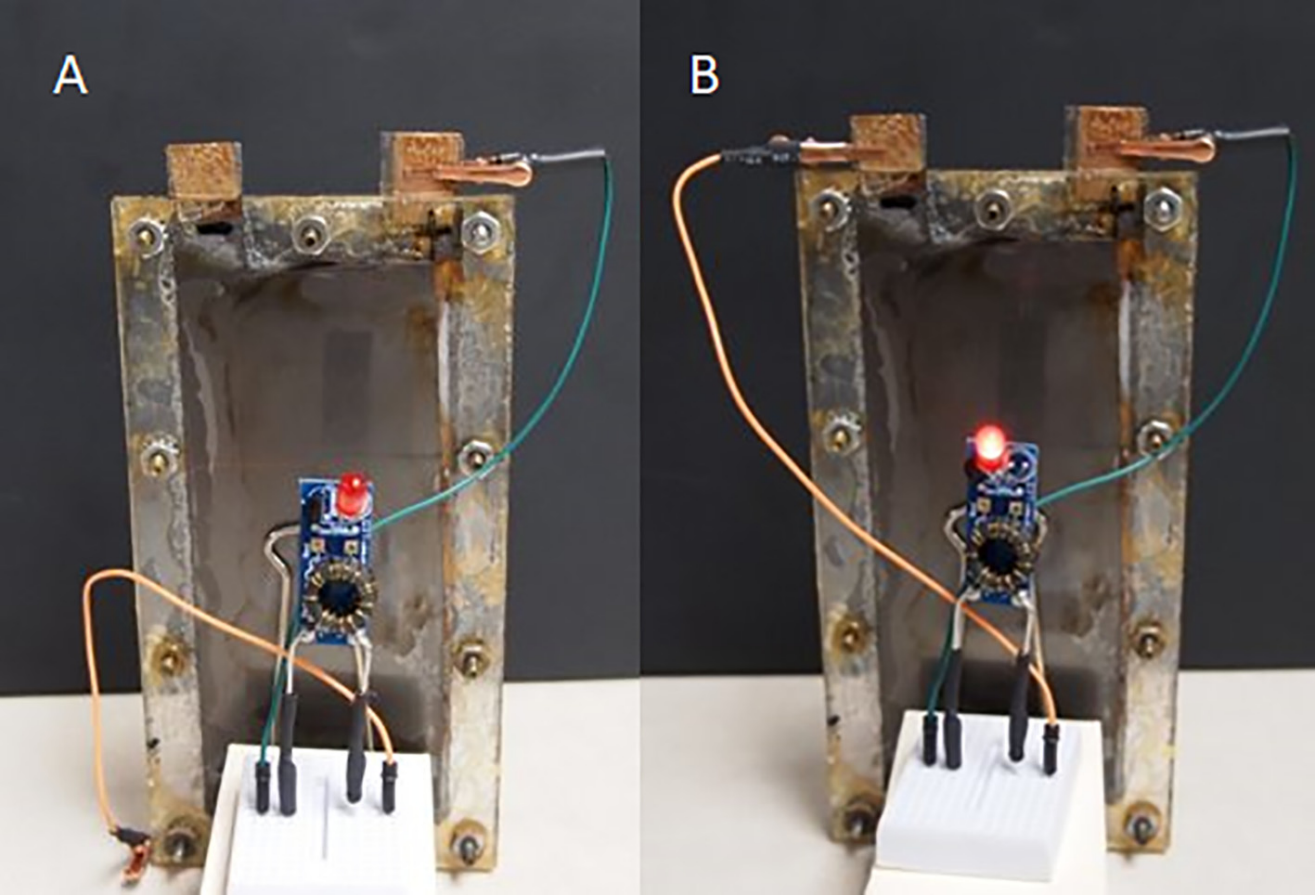
Iron battery 2.0 and Joule thief power an LED. (A) Photograph shows open circuit (LED off). (B) Photograph shows closed circuit (illuminated LED).

As one example application, we used a single cell with a “Joule thief” circuit to boost the voltage. This circuit converts the low voltage DC to AC and drives the AC voltage through a step-up transformer. The result is sufficient for a modest brightness LED (see Fig. 5). As an alternative, five such batteries could be wired in series. They could act as a storage reservoir for solar energy to operate a light at night. This battery may represent some advantages over lithium ion batteries including lower cost and lower environmental impact of production as well as low toxicity and high recyclability.

The iron battery has significant internal resistance. Beyond its maximal current, the internal resistance results in a significantly lower operational voltage. The lower voltage reduces the available power. The maximal energy density for the 8 ml cell was 0.25 mW per ml. A battery should be designed and constructed with this limitation in mind. Depending on power requirements, a battery of the appropriate size will be needed.

During the preparation of reagents, there are several safety considerations. Potassium hydroxide is caustic and should not come into contact with skin. Ferric chloride is corrosive and strongly acidic and should not be allowed to come into contact with skin. Additionally, ferric chloride will degrade metals it contacts. Once the components are neutralized by mixing in the proper proportions, they are comparatively inert. When the cell is no longer needed, it can be considered non-hazardous waste (subject to local regulations). The plastic, neutral pH water, iron salts, and potassium salts are all non-hazardous waste (comparable to normal food and household waste).

## 7 Validation and characterization

**Fig. 6:**
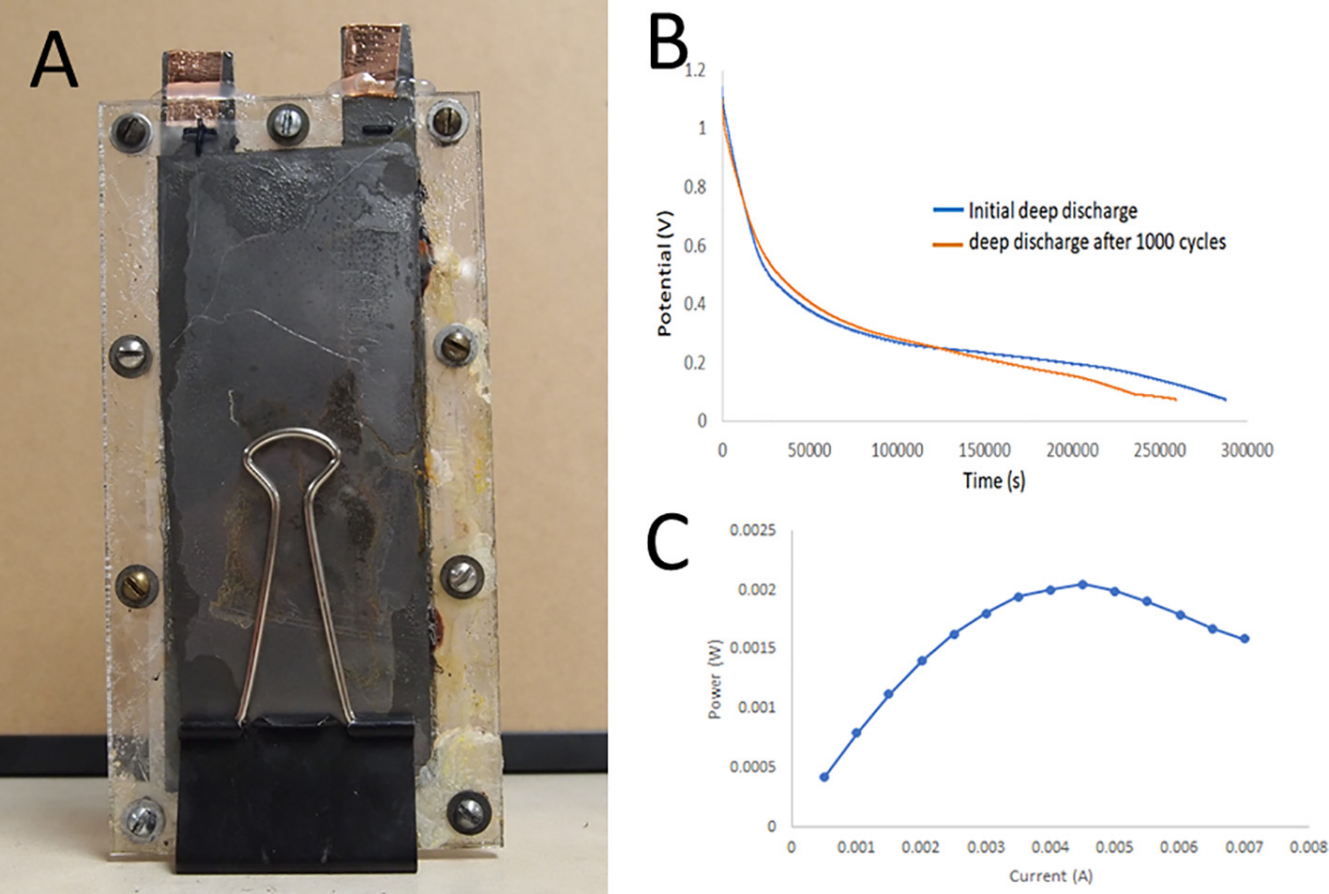
Cell characterization. (A) Photograph of assembled cell. (B) Graph shows deep discharge before and after 1000 cycles. (C) Graph of power vs current shows maximal power.

The 8 ml version of the iron cell was tested for its total capacity and maximal power. The cell is shown in Fig. 6A. This cell contains 4 ml each of anode and cathode paste. It has a membrane of 48 cm^2^. To determine the total capacity, the cell was discharged at a constant current of 1 mA until the potential dropped to 75 mV as shown in Fig. 6B. The Total capacity on first discharge was 80 mAh. With 0.97 g of ferric iron acting as a cathode, we would expect a maximum of 160 mAh. Thus, we are accessing 50% of the iron in the cathode. After 1000 cycles, the capacity was re-measured and was 72 mAh, showing a loss of only 10%.

To determine the maximal power, the 8 ml cell was discharged at a range of currents and the voltage was measured. The power was calculated at each condition by multiplying the current and voltage. The result was a graph of power as a function of current, as shown in Fig. 6C. The maximal power was ~ 2 mWatts or 0.25 mW/ml at 4.5 mA. This is significantly lower than typical commercial batteries, but nearly two orders of magnitude larger than our previous best.

Capabilities and limitations:

• Voltage: Although the cell shows an open cell potential of 1.1 V, the voltage may drop as low as ~ 200 mV during high current discharge. This internal resistance reduces available power.
• Volumetric Capacity: 10 Ah/L. This limits the battery to stationary applications.
• Energy density: >3 Wh/L
• Power density: 250 mW/L
• Cycle stability: Stable for greater than 1000 cycles
• Price: $60 per watt-hour for 8 ml cells (as built, including laser-cut housing)
• Price: $0.36 per watt-hour for active materials at wholesale prices

Human and animal rights

This work did not use human or animal subjects.

## Declaration of Competing Interest

The authors declare that they have no known competing financial interests or personal relationships that could have appeared to influence the work reported in this paper.
