# Supplementary material for: Open source all-iron battery 2.0
Source: HardwareX. 2021 Jan 2;9:e00171. doi: 10.1016/j.ohx.2020.e00171 (PMC9041250; doi:10.1016/j.ohx.2020.e00171)
Supplement: Supplementary data 1 [file mmc1.docx]

**Supplemental Build Instructions: Open Source All-Iron Battery 2.0**

**Authors: Dipak Koirala, Nicolas Yensen, Peter B. Allen***

**Affiliations: University of Idaho**


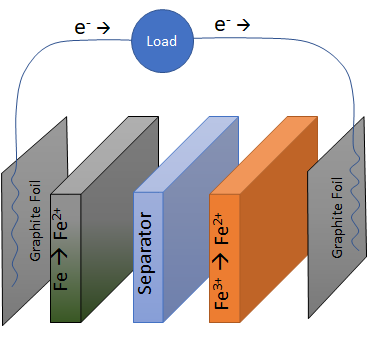


***Battery Schematic***

**1. Laser-cut Cell Housing**

Acrylic sheets [Housing] were laser cut per CAD drawings (available at repository [*https://osf.io/6z8ny/*](https://osf.io/6z8ny/)). Acrylic plastic of desired thickness can be purchased from a hardware store or online as Plexiglass brand or similar. As an alternative, the sheets can be ordered pre-cut from a commercial laser cut prototyping service. 1 mm thick acrylic (poly methyl methacrylate, PMMA) was used. Any plastic or paper backing should be removed from the laser cut sheets before use.

The included design will generate a cell with an enclosed volume of 9.6 ml. This cell will produce 1 V – 1.2 V and hold 80 mAh. The dimensions can be altered to enclose a larger or smaller volume. Thicker and thinner plastic may change the performance characteristics such as maximal current per unit volume.

**2. Chemical Solutions**

**2.a. Ketjen Black [Conductive Carbon]**

20 g of Ketjen black EC-600JD was ball-milled for ~24 hrs in United Nuclear Ball Mill (3 Lb. Capacity) to obtain fine particles with large surface area.

**2.b. 15 M KOH [Base]**

10 ml of 15 M KOH solution was prepared by dissolving 8.42 g of KOH pallets in about 6 ml of DI water in plastic container (centrifuge tube).

*Caution: The container being used will become hot to the touch and is a burn hazard. Exercise care when handling and once KOH is completely dissolved, allow it to return to room temperature before handling.* *Gloves and goggles are necessary.*

Water was added up to the mark to bring final volume to 10 ml.

**2.c. Cathode electrolyte**

Cathode paste electrolyte consists of ~2 M FeCl_3_ [Fe Salt 2] in 2 M K_2_SO_4_ [Salt] at 7.5 pH. Here, 3.49 g of K_2_SO_4_ was added to 5.5 ml of DI water taken in a beaker and slowly stirred. Then 3.24 g of FeCl_3_ was slowly added to above mentioned solution and stirred for about 5 minutes at room temperature. 4.34 ml of 15 M KOH was added dropwise and stirred until the solution cools down to room temperature. Few drops of KOH or HCl was added to maintain pH 7.5. Finally, 0.60 gm of Ketjen black (conductive carbon) was added and mixed (with help of mortar and pestle) to form a homogenous paste.

Depending on the purity and accuracy of the KOH solution, the precise volume to reach pH 7.5 may be different. This can be determined by titration as with ferric chloride. This can be accomplished as follows:

- Weigh ~1 g of the FeCl_3_ / water / K_2_SO_4_ mixture in a small plastic container and record the mass. Dilute this with ~20 ml of water (this volume is arbitrary but should be just enough to immerse a pH probe). Add as stir bar and stir plate. (See Schematic below)
- A dilute KOH solution (~1.5M) should then be prepared by adding 5 ml of 15 M KOH and adding sufficient water to reach 50 ml. This dilute KOH can be used as a titrant.
- This dilute KOH solution is then added dropwise from a burette while monitoring with a pH probe. When the solution reaches pH 7.5, the total volume of dilute KOH delivered should be noted.


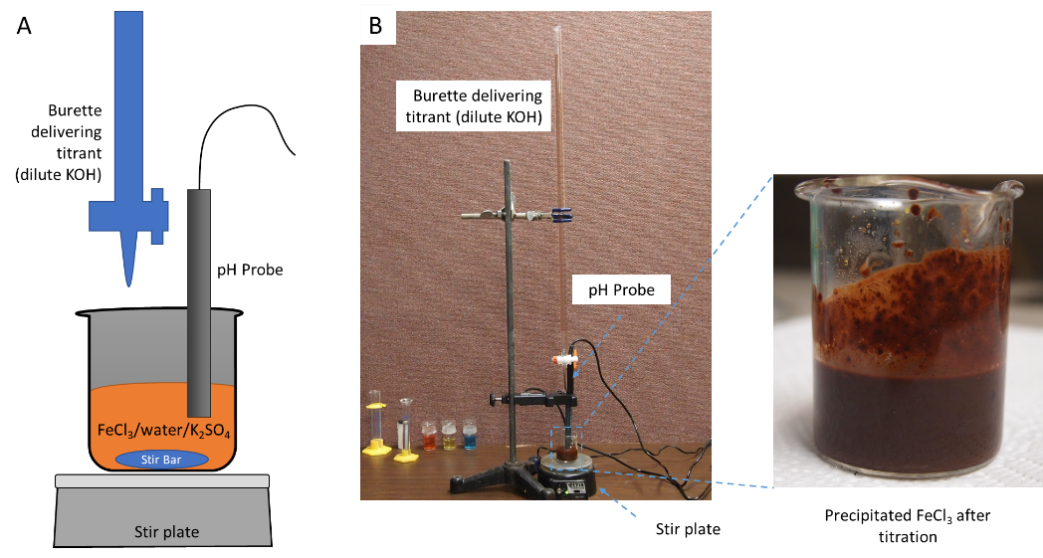


Figure S.1: Illustration of titration. (A) Schematic shows how burette and pH probe are positioned in the dilute mix of ferric or ferrous chloride and a stir bar. The burette is positioned such that it delivers drops of dilute KOH into the solution. (B) Image shows the apparatus set up in the lab. Zoomed image shows the case of the ferric chloride after titration to pH 7.5.

The ratio of KOH to solid FeCl_3_ /water / K_2_SO_4_ can then be calculated. For example, if 1.14 g of the FeCl_3_ / water / K_2_SO_4_ mix was used for the titration, and this required 4.05 ml of ~1.5 M KOH, then a weighed 12.23 g of FeCl_3_ / water / K_2_SO_4_ should be treated with 4.34 ml of 15 M KOH.

Sample calculation:

| 3.24 | g FeCl_3_ |  |  |  |  |
| --- | --- | --- | --- | --- | --- |
| 5.5 | g water |  |  |  |  |
| 3.49 | g K_2_SO_4_ |  |  |  |  |
| 12.23 | g total FeCl_3_ / water / K_2_SO_4_ | | |  |  |
|  |  |  |  |  |  |
| 1.14 | g (measured) FeCl_3_ / water / K_2_SO_4_ | | | |  |
| 4.05 | ml (measured) 1.5 M KOH to reach pH 7.5 | | | | |
|  |  | |  |  |  |
| 3.55 | ml 1.5 M KOH per gram FeCl_3_ / water / K_2_SO_4_ | | | | |
| 0.355 | ml 15 M (concentrated) KOH per gram FeCl_3_ / water / K_2_SO_4_ | | | | |
|  |  |  |  |  |  |
| 11.09 | g (measured) remaining FeCl_2_ / water / K_2_SO_4_ | | | |  |
| 3.93 | ml of 15 M KOH is needed to reach pH 7.5 | | |  |  |

The percentage composition of cathode paste was as follows;

| Compound | Weight % |
| --- | --- |
| FeCl_3_ | 16.88 |
| K_2_SO_4_ | 18.18 |
| H_2_O (density = 0.998 g/ml) | 28.58 |
| 15 M KOH (in H_2_O) (density = 1.47 g/ml) | 33.23 |
| Ketjen Black | 3.13 |

*Caution: FeCl_3_ is a strong Lewis acid. The pH will be below 0 for a 2M solution. Care must be exercised during handling. Gloves and proper PPE are recommended.*

**2.d. Anode electrolyte**

The anode electrolyte paste preparation follows the same pattern as the cathode electrolyte. This paste consists of ~2M FeCl_2_.4H_2_O [Fe Salt 1] in 2M K_2_SO_4_ [salt] at pH 7.5.

3.98 g of FeCl_2_.4H_2_O was weighed and transferred in a beaker and 7.8 ml DI water is added to it. Next 3.49 g of K_2_SO_4_ was weighed and transferred to the above solution and the solution was stirred for 10 min at room temperature followed by slow addition of 2.28 ml of 15M KOH solution. Final volume of Anolyte was ~12.5 ml (only 4 mL was used). The pH of the solution was measured using a glass pH electrode and increased to pH 7.5 by dropwise addition of KOH. Depending on the purity and accuracy of the KOH solution concentration, the precise volume required to reach pH 7.5 may be different. This can be determined by titration.

The appropriate quantity of 15 M KOH can then be added to reach pH 7.5. Finally, 0.60 gm of Ketjen black (conductive carbon) was added and mixed (with help of mortar and pestle) to form a homogenous paste. The percent composition of anolyte of our preparation was as follows:

| Compound | Weight % |
| --- | --- |
| FeCl_2_.4H_2_O | 20.75 |
| K_2_SO_4_ | 18.19 |
| H_2_O (density = 0.998 g/ml) | 40.46 |
| 15 M KOH (in H_2_O) (density=1.47 g/ml) | 17.47 |
| Ketjen Black | 3.13 |

**3. Cell Materials Preparation**

Once the solutions are prepared, the cell can be prepared. This includes the cell housing, membrane, steel wool anode [Fe Metal], graphite foil current collectors [Ele1]. The cell can be assembled once the parts are prepared.

**3.a. Membrane Preparation**

A4 printing paper was cut in circular piece of diameter 19.55 cm (300 cm^2^ area).

600 mg (2mg/cm^2^) of cellulose acetate was weighed and transferred in a conical flask and 30 ml (0.1ml/cm^2^) of acetone was added to it. Then 300 μL (1 μL/cm^2^) of 5% Nafion suspension and 450 μL (1.5 μL/cm^2^) of ethylene glycol was added and the flask was tightly corked and it was stirred at room temperature for ~12 hours. We note that Nafion can be omitted and the battery will still function, albeit at higher self-discharge rates.

The mixture was poured over the printing paper and was left to evaporate overnight at room temperature.

**Figure S.2:** Membrane Preparation. (A) Circular glass casting tray and a sheet of printing paper. (B) Printing paper cut in required area to fit the circular area of tray. (C) Casting the mixture over paper. (D) Paper membrane ready to use.

**3.b. Steel Wool Preparation**

Steel wool was used in anode of the cell. 0.45 g of steel wool [Fe Metal] was weighed and finely chopped with scissors into ~2 mm length pieces.

**3.c. Graphite Foil Preparation**

The graphite foil [Ele1] acts as the current collector for the anode and the cathode. It electrically connects the anode and cathode of each cell. Two strips with dimensions of 15 cm x 5 cm (thickness ~ 0.1-0.3 mm) were cut to shape with a sharp cutting tool (utility knife or scissors). Copper foil was attached to one end to physically reinforce the foil and make a solid electrical contact (see Photo 1 in Figure S.3 below).

**4. Cell Housing Assembly**

The laser cut acrylic from Section 1 was assembled along with silicone adhesive, graphite foil, and electrode pastes. Adhesive (liquid nails [Glue] or other waterproof caulk) was applied in the interior side (Figure S.3 step 1). Then the graphite foil was applied to the adhesive (step 2). Further adhesive was then applied to the graphite to generate a seal to the second layer of acrylic (step 3). The graphite is thus sandwiched between the sheets of acrylic in contact with the glue (step 4). The acrylic-glue-graphite-glue-acrylic sandwich assembly was aligned and held together position with binder clips for ~12 hrs to ensure that the glue was fully cured. Copper foil was added to the graphite to reinforce it for mechanical connection with alligator clips (step 5). Two such assemblies were constructed, one each for anode and cathode (steps 6-10).


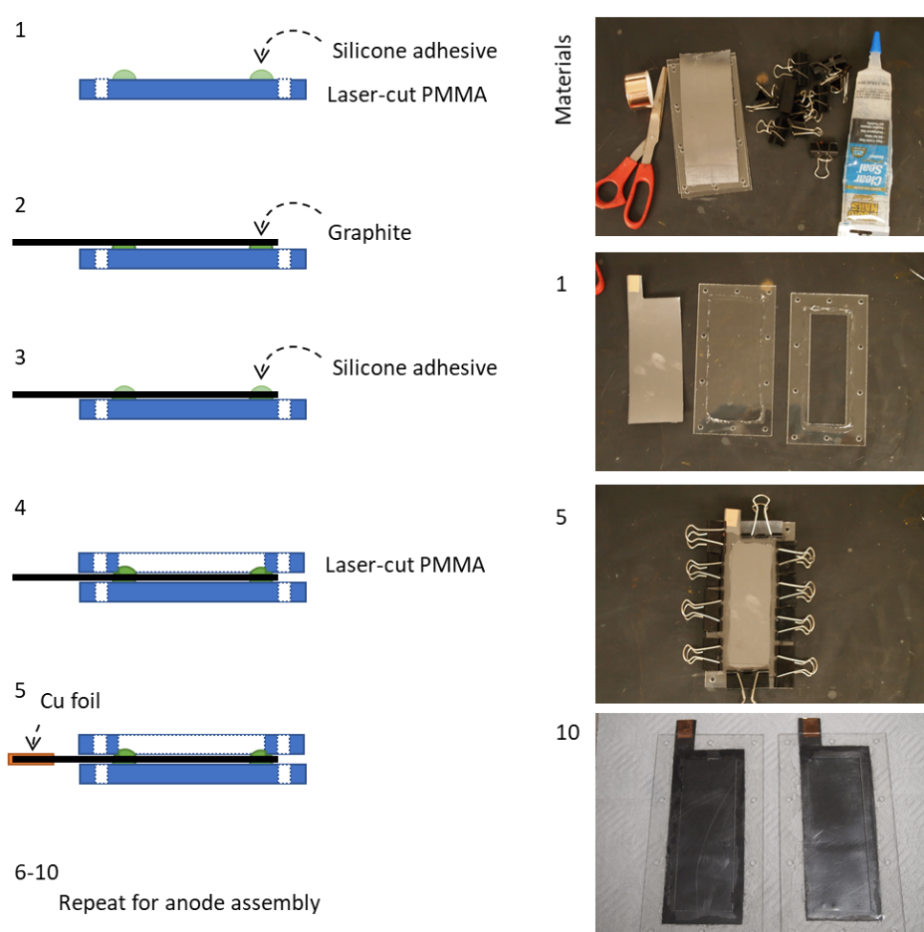


*Figure S.3: Cell housing assembly. Left shows schematic of the assembly process through the numbered steps. Images at right show images of the materials and selected steps of the process.*

**5. Battery Cell Final Assembly**

One finished half-cell housing was taken and labelled as cathode. ~4ml of catholyte paste was added and evenly distributed in the void space (Figure S.4 step 11).

Similarly, another half-cell housing was assembled as above. This should be labelled as anode. 0.45 g of steel wool was added to the void space of it and ~4 ml of anolyte paste was evenly distributed in the void space (step 12).

A small amount of silicone adhesive was applied on the inner boundary of the cathode cell (step 13) and a pre-cut 13.5 cm ×5.4 cm membrane was kept on the top of it (step 14).

Again, a small amount of silicone adhesive was applied on the boundary of the membrane (step 15) and the anode half cell was carefully place on the top of cathode half-cell with membrane (step 16) and held in place with binder clips. Once the adhesive cured, the assembly was fitted with screws.


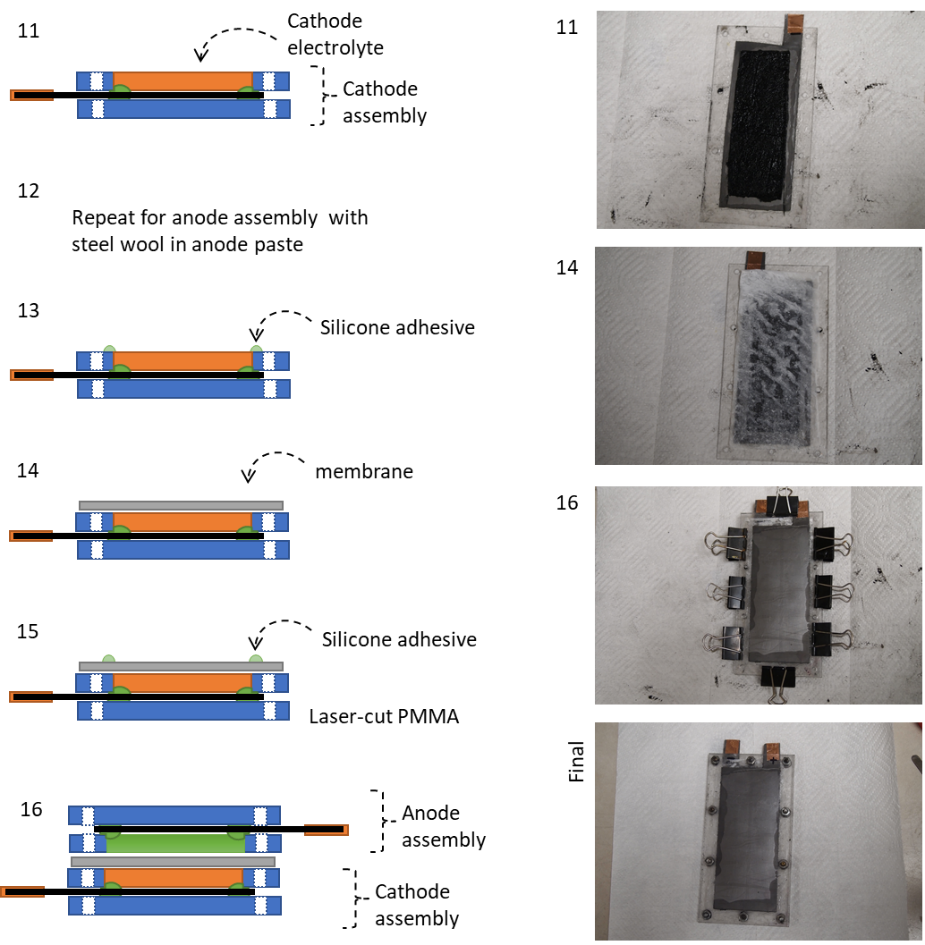


Figure S.4: Assembly of Cell. Left shows schematic of the assembly process through the numbered steps. Images at right show images of the materials and selected steps of the process and the final, assembled cell.
